# Mutant glucocorticoid receptor binding elements on the interleukin-6 promoter regulate dexamethasone effects

**DOI:** 10.1186/s12865-021-00413-z

**Published:** 2021-03-26

**Authors:** Wen-Teng Chang, Ming-Yuan Hong, Chien-Liang Chen, Chi-Yuan Hwang, Cheng-Chieh Tsai, Chia-Chang Chuang

**Affiliations:** 1 Department of Biological Science and Technology, Chung Hwa University of Medical Technology, Tainan, 701 Taiwan; 2 Department of Emergency Medicine, National Cheng Kung University Hospital, College of Medicine, National Cheng Kung University, Tainan, Taiwan; 3 Department of Physical Therapy, I-Shou University, Kaohsiung, Taiwan; 4 Department of Nursing, Chung Hwa University of Medical Technology, Tainan, 701 Taiwan

**Keywords:** Glucocorticoid receptor, Interleukin-6, Lipopolysaccharide, Dexamethasone, Promoter activity, Transcriptional factor

## Abstract

**Background:**

Glucocorticoids (GCs) have been extensively used as essential modulators in clinical infectious and inflammatory diseases. The GC receptor (GR) is a transcription factor belonging to the nuclear receptor family that regulates anti-inflammatory processes and releases pro-inflammatory cytokines, such as interleukin (IL)-6.

**Results:**

Five putative GR binding sites and other transcriptional factor binding sites were identified on theIL-6 promoter, and dexamethasone (DEX) was noted to reduce the lipopolysaccharide (LPS)-induced IL-6 production. Among mutant transcriptional factor binding sites, nuclear factor-kappa B (NF-κB), activator protein (AP)-1, and specificity protein (Sp)1–2 sites reduced basal and LPS-induced IL-6 promoter activities through various responses. The second GR binding site (GR2) was noted to play a crucial role in both basal and inducible promoter activities in LPS-induced inflammation.

**Conclusions:**

We concluded that selective GR2 modulator might exert agonistic and antagonistic effects and could activate crucial signaling pathways during the LPS-stimulated inflammatory process.

**Supplementary Information:**

The online version contains supplementary material available at 10.1186/s12865-021-00413-z.

## Background

Severe sepsis is related to immune dysequilibrium. Immune dysregulation during the early phase of sepsis results from the inadequate endogenous glucocorticoid (GC)-mediated regulation of nuclear factor-κВ (NF-κВ) activation, which leads to its overexpression and release of massive proinflammatory cytokines (the so-called “cytokine storm”) [1]. Notably, “GC sensitivity” in critically ill patients with septic shock is associated with disease severity and outcome [2]. Although low-dose steroid administration during the early septic phase could not reduce 28-day mortality, it could reduce the duration of shock and mechanical ventilator dependency [3–5]. However, the effects of low-dose steroids on the short- and long-term mortality of patients with sepsis remain controversial. GCs regulate several biological processes through their intracellular GC receptors (GRs). Notably, GCs diffuse through the cell membrane and bind to their GRs. The activated GR complex then translocates into the nucleus and expresses inflammatory mediators [6].

GCs modulate the innate and adaptive immune systems responding to infection in mammals. However, the actual mechanism of change in transcriptional level of GR remains unclear. Toll-like receptors (TLRs) are well-known pattern-recognition receptors and are responsible for early detection of various microbial pathogens [7]. Activation of TLRs initiates intracellular target gene transcription and GCs could suppress TLR-mediated signaling transduction [8]. The mechanism of GCs suppression on TLR is supposed to either through glucocorticoid-induced leucine zipper (GILZ) inhibitors or through NF-κВ, activator protein-1 (AP-1) complexes inhibition [8]. The activation of TLR2 on epithelial cells will increase the secretion of IL-6 and IL-8. Moreover, the activation of both TLR2 and 4 within the adrenal gland could also lead to the cortisol and corticosterone production [8]. The crosstalk between GRs and TLR signaling, and the cross-utilization of cofactors essential for both GRs and TLRs are complex. The TLRs signaling networks seem to be an important role in modulating innate immunity and other immune responses [7, 8].

GCs are considered immunosuppressive and anti-inflammatory agents and used as adjuvant therapy in patients with severe sepsis and septic shock. Activated GRs interact directly and indirectly with promoters to negatively regulate cytokine gene expression [9, 10]. The selective knockout of the GR gene results in sensitivity to lipopolysaccharide (LPS) treatment, whereas endogenous GCs reduce LPS-induced inflammation by inhibiting cytokine gene expression, such as that of interleukin (IL)-12 [11]. In addition, GCs negatively regulate IL-6 gene expression through the downregulation of its promoter activity in various tissues [12, 13]. The regulation of the gene expression of several cytokines by activated GRs, which bind to GCs, may occur through binding to genes or indirect interaction with other transcription factors, particularly NF-κB and AP-1 complexes [9, 14–16]. Transcription factors, such as NF-κB and AP-1, extensively mediate gene expression during the inflammatory reaction, especially in inflammatory cytokines, matrix metalloproteinases (MMPs), and cyclo-oxygenase (COX)-2 [14, 17, 18]. Dexamethasone (DEX), a synthetic GC, was reported to inhibit the inflammatory response by downregulating the transcription factor AP-1 in human lung epithelial cells [18].

We previously demonstrated an increase in the macrophage migration inhibitory factor (MIF) in severe sepsis conditions, such as in the *Vibrio vulnificus-*infected model [19]. In *V. vulnificus*-infected mice, MIF could regulate IL-6 in a time-dependent manner. Serum MIF regulates NF-κB to modulate the production of IL-6 at the transcriptional level. In the present study, we investigated the mechanisms of transcription factors involved in regulating the IL-6 promoter. In addition, we elucidated the roles of putative transcriptional factor binding sites, such as NF-κB, specificity protein (Sp) 1, and AP1, in the LPS-induced sepsis model. Furthermore, we examined the causal link between GRs and pro-inflammatory transcription factors.

## Methods

### Cell culture

Two cell lines were used in the present study. One was the RAW 264.7 mouse macrophage cell line purchased from Culture Collection and Research Center, Food Industry and Development Institute, Hsinchu, Taiwan. The cells were grown in Dulbecco’s modified Eagle medium (Sigma) supplemented with 10% fetal bovine serum (Gibco Laboratories, Grand Island, NY) in humidified atmosphere containing 5% CO_2_ at 37 °C. The second cell line was human neuroblastoma IMR-32 cells, obtained from the American Type Culture Collection (ATCC), which were cultured in minimum essential medium (MEM) (Genedire X, USA) supplemented with 10% fetal bovine serum and 1% sodium pyruvate at 37 °C in a controlled atmosphere of 5% CO_2_. After 2- to 3-day growth that filled up to 70–80% of a 10-cm culture dish, the IMR-32 cultures were subcultured or collected, and nuclear proteins were extracted for performing the electrophoretic mobility shift assay (EMSA).

### Chemicals

LPS (*Escherichia coli* O111: B4) was purchased from Calbiochem® (Detroit, MI). DEX and inhibitors were purchased from Sigma Chemical Co. (St. Louis, MO).

### Measurements of IL-6 expression

The mRNA levels of IL-6 were measured using the semi-quantitative RT-PCR. Total RNA was isolated from the cultured cells by using TRIzol reagent (Invitrogen). RT-PCR was performed as described previously (Chang and Huang, 2005). Briefly, total RNA (2 μg) was reverse-transcribed into cDNA in 20 μL of 1× first strand buffer containing 0.5 μg of oligo (dT) as a primer, 500 μM dNTP, and 200 units of SuperScript II (Invitrogen). PCR was performed in 20 μL of 1× PCR buffer containing 2 μL of RT products, 1 unit of AmpliTaq DNA polymerase (Roche Applied Science), 200 μM dNTP, and 1.5 mM MgCl_2_ (Amersham Biosciences), and 0.4 μM primer pair. We used the primer pair of IL-6 mRNA, mIL6-mF1, and mIL6-mR1 (presented in Supplemental information). The PCR parameters were 94 °C for 30 s, 53 °C for 30 s, and 72 °C for 30 s for 30 cycles, followed by a final elongation at 72 °C for 7 min. PCR products were analyzed on 1.2% agarose gel.

The concentrations of IL-6 in media were assayed using ELISA MAX Standard Sets (BioLegend, CA, USA) according to the manufacturer’s instructions.

### Plasmid constructs

The pGL4.10-basic and pRL-TK luciferase reporter vectors (Promega, Madison, WI) were used for the promoter reporter assay. The promoter construct of the IL-6 gene was generated through PCR amplification of a 349-bp fragment (− 364/− 15, Supplemental information) that was then inserted with the KpnΙ/HindIII fragments of the human IL-6 gene promoter into the pGL4.10-Basic vector. The resulting plasmid was named pGL4.10-IL-6 construct. Promoter constructs containing nucleotide substitutions in the sequence motifs of AP1, NF-κB, Sp1, and five GR sites were individually generated using PCR amplification with primer pairs spanning the mutant nucleotides according to the protocol of site-directed mutagenesis through overlap extension [20]. The sequences of primers used in cloning the IL-6 promoter and site-directed mutagenesis are listed in supplemental information.

### Transient transfection

Exponentially growing RAW 264.7 cells were seeded at a density of 2.0 × 10^5^ cells per well in 12-well plates. After 24 h, transient transfection was performed using PolyJet DNA transfection reagent (SignaGen Laboratories, Rockville, MD, USA) per the manufacture’s protocol at a transfection reagent to DNA ratio of 1:3. The plasmid DNA was then extracted and purified using the UltraClean endotoxin removal kit (MoBio Lab, Carlsbad, CA) before co-transfection. The plasmid mixtures containing 0.5 μg of pGL4.10-IL-6 plasmid and 0.5 μg of pGL4.74 [*hRluc*/TK] vector were co-transfected for 24 h. After the medium was changed, LPS (1 μg/mL) or DEX (10 μM/mL) were added to the plates.

### Dual luciferase assay

After 24 h of LPS (1 μg/mL) or DEX (10 μM/mL) treatment, the cells were washed with PBS, and then, lysates were prepared by scraping the cells from plates in the presence of 1× passive lysis buffer (Promega). The luciferase assay was performed using the dual luciferase assay system (Promega) and then detected using the Sirius luminometer (Berthold Detection System, Pforzheim, Germany).

### Nuclear protein extraction of human IMR-32 cells

IMR-32 cells were collected from the culture dish and centrifuged for 5 min at 500×g. The cell pellets were mixed with a 10-fold volume of buffer A (10 mM HEPES [pH 7.9], 1.5 mM MgCl_2_, 10 mM KCl, 0.5 mM DTT, and one tablet of protease and phosphatase inhibitor [Thermo Scientific, USA]) and incubated on ice for 8 min followed by short centrifugation for 10 s at 12,000×g at 4 °C. The buffer A-treated cell pellets were then mixed with a two-fold volume of buffer C (20 mM HEPES [pH 7.9], 25% glycerol, 420 mM NaCl, 1.5 mM MgCl_2_, 0.2 mM EDTA, 0.5 mM DTT, and one tablet of protease and phosphatase inhibitor) and incubated on ice for 16 min. After centrifugation at 12,000×g at 4 °C, the supernatant containing the nuclear protein of IMR-32 cells was collected and stored at − 80 °C.

### Gel electrophoretic mobility shift assays

During EMSA, DNA probes with putative GR-binding sites were prepared by annealing the two complementary biotin-labeled single-strand oligonucleotides as follows: an equal amount of complementary oligonucleotides was mixed in 1× DNA-binding buffer and heated at 94 °C for 2 min and then gradually cooled down to 25 °C. In this study, EMSA was performed using the LightShift® Chemiluminescent EMSA kit (Thermo Scientific, USA) according to manufacturer’s instructions. The nuclear protein from IMR-32 cells was first incubated with 62.5 ng of poly (dI-dC)•poly (dI-dC) at 4 °C in the 1× binding buffer for 30 min to reduce the possibility of nonspecific binding. For competition experiments, excess of unlabeled DNA was added in the binding reaction. The biotin-labeled DNA probes were then added and incubated at 4 °C for 30 min. Approximately 20 μL of mixtures were electrophoresed on a 4% native gel at 100 V and then transferred onto a 0.45-μM positive-charged nylon membrane (Pall Corporation, USA) at 380 mA in cold 0.5× TBE buffer (VWR International, USA). Bands on the membrane were visualized using the streptavidin–HRP hybrid, followed by enhanced chemiluminescence treatment.

### Chromatin immunoprecipitation assay [21]

Chromatin immunoprecipitation (ChIP) assays were performed using an EZ ChIP kit purchased from Upstate Biotechnology (Millipore Corporation Billerica, MA). Briefly, RAW 264.7 cells were fixed with 1% formaldehyde for 10 min at room temperature for DNA cross-linking. Chromatin was sheared through sonication under optimized conditions. ChIP reactions were conducted according to the manufacturer’s protocol with anti-GR, anti-RNA polymerase II, and anti-mouse IgG (Santa Cruz, CA). The IL-6 promoter region was amplified using the primer pair mIL-6 F1 and mIL-6 R3. PCR cycle parameters were as follows: 41 cycles of 95 °C for 20 s, 58 °C for 30 s, and 72 °C for 30 s, and a final extension for 15 min at 72 °C. PCR products were separated using agarose gel electrophoresis.

### Statistical analyses

All values are expressed as the means ± standard deviation (SD). An intergroup comparison was performed using Student’s two-tailed unpaired *t*-test. Statistical evaluation was performed by the statistical software SPSS 17.0. *P* < 0.05 was considered statistically significant.

## Results

### LPS-induced morphological changes in RAW 264.7 and its IL-6 gene expression was reversed by DEX co-treatment

**Fig. 1:**
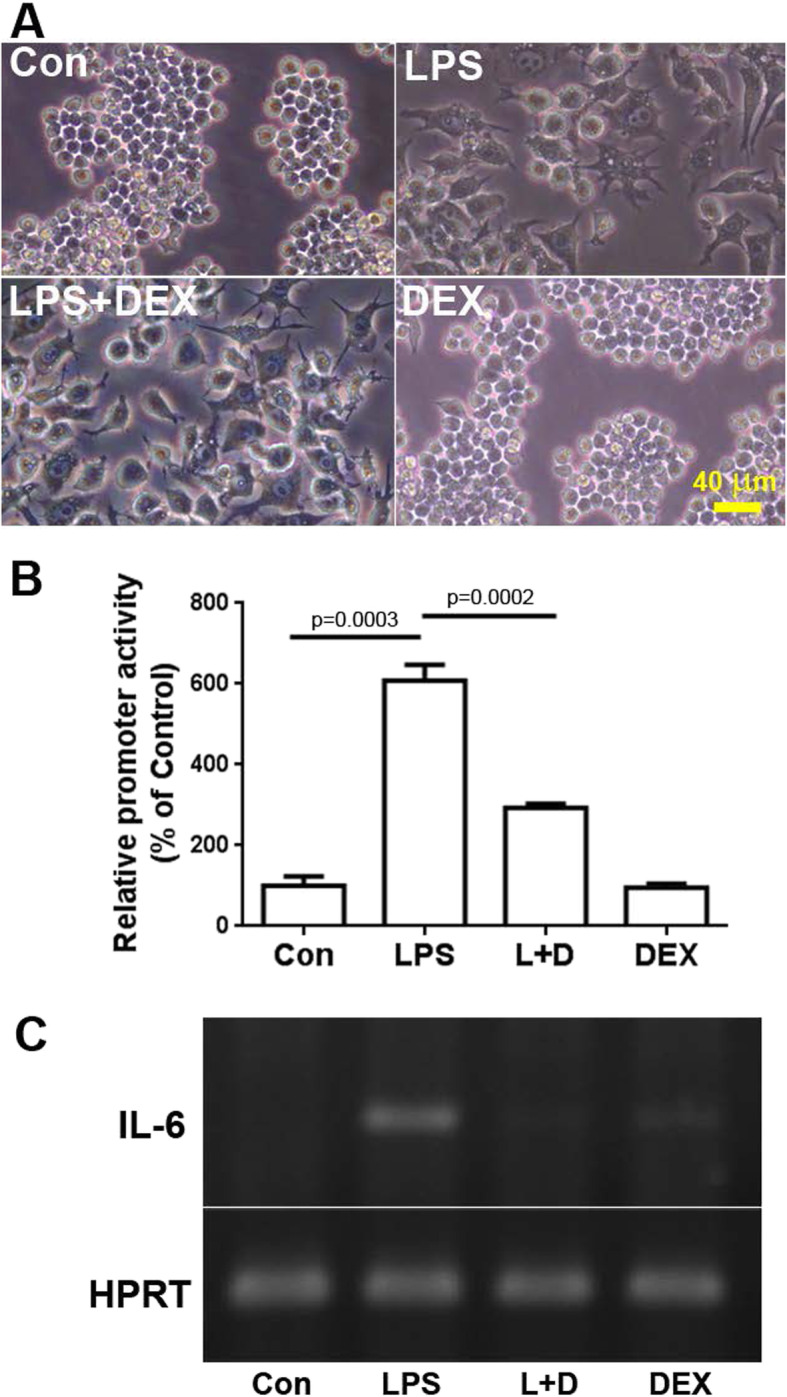
Dexamethasone (DEX) downregulates lipopolysaccharide (LPS)-induced macrophage activation and IL-6 gene expression. **a** Morphological changes were induced by LPS and DEX. RAW 264.7 cells were treated with 1 μg/mL of LPS and 10 μM DEX for 24 h. **b** DEX mediated the LPS-induced IL-6 promoter activities. **c** The mRNA levels of IL-6 were induced by LPS and reversed by adding DEX. All values are expressed as the mean ± SD and each experiment was conducted with triplicate cultures. The intergroup comparisons were performed using Student’s two-tailed unpaired *t*-test

The RAW 264.7 cells were treated with LPS and DEX, and the promoter activities and RNA levels of IL-6 gene were measured to determine the LPS-induced effects of GCs on IL-6 gene expression. We observed that the morphology of RAW 264.7 cells was changed under LPS treatment to perform macrophage-like activities and accompanied the cytokine gene expression, such as IL-6 (Fig. 1a). In addition, to study IL-6 gene regulation, we constructed a 349-bp promoter fragment of IL-6 gene into the pGL4.10-basic vector, which contains a reporter luciferase gene for measuring promoter activity. Additionally, RT-PCR was used to detect the IL-6 gene transcript. We noted that the promoter activities and mRNA levels were induced by LPS and reversed by DEX (Fig. 1b and c). However, DEX treatment alone did not alter the cellular morphology, promoter activities, and mRNA levels of IL-6 gene. These results indicate that GCs modulated the LPS-induced morphological change and IL-6 gene expression.

### Different changes in promoter activities in mutant putative sites on the IL-6 promoter assessed through site-directed mutagenesis

**Fig. 2:**
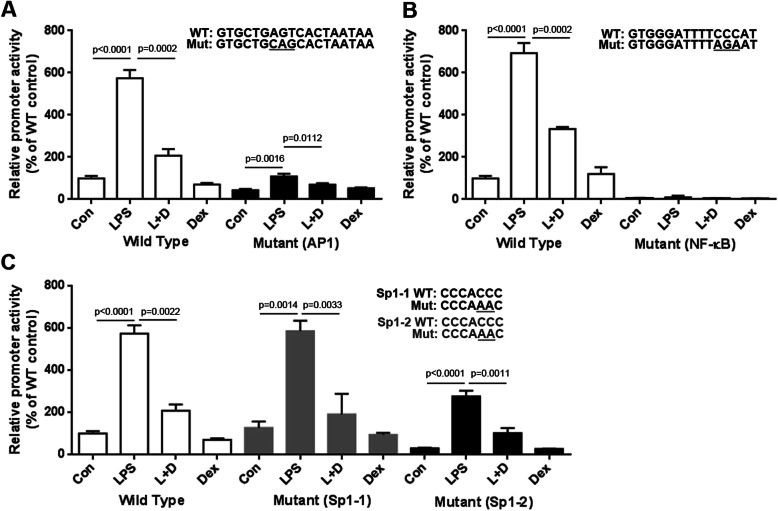
Effects of mutation of the putative transcriptional factor binding sites. **a** Promoter activities of mutant AP-1 sites were significantly decreased compared with wild-type controls. **b** Promoter activities of mutant NF-κB sites were more significantly decreased than wild controls. **c** Promoter activities of mutant Sp1–2 sites revealed a partially decreased change compared with wild-type controls; however, no significant differences were noted between mutant Sp1–1 sites and wild-type controls. All values are expressed as the mean ± SD and each experiment was conducted with triplicate cultures. The intergroup comparisons were performed using Student’s two-tailed unpaired *t*-test

We searched the transcriptional factor binding sites on the IL-6 promoter region by using bioinformatics tools to determine proteins that bind to the IL-6 promoter to mediate gene expression and noted several putative sites, such as AP-1, NF-κB, Sp1, and five GR sites (GR1, GR2, GR3, GR4, and GR5). The sequences of these sites revealed high similarity among different species (Supplemental material, Fig. S1). Furthermore, we used site-directed mutagenesis to construct the mutant IL-6 promoter-reporter vectors and transfected the RAW 264.7 cells with these constructs to identify the sites that are crucial for regulating IL-6 gene expression. The mutation of the AP-1 site reduced the basal and LPS-induced effects, as well as the effects of DEX addition on IL-6 promoter activities (Fig. 2a). Moreover, the mutation of the NF-κB site dramatically reduced promoter activities (Fig. 2b). Notably, two Sp1 sites are present on the IL-6 promoter, denoted as Sp1–1 and Sp1–2. The mutation of the Sp1–2 site but not the Sp1–1 site reduced IL-6 promoter activity, suggesting that these two sites exert differential function in IL-6 expression in this cell line (Fig. 2c). These results indicated that AP-1 and NF-κB sites are crucial for IL-6 expression in RAW 264.7 cells, but only one Sp1 site was involved.

### Basal and inducible changes in IL-6 promoter activities in five mutant GR binding sites and the reversed effect of DEX treatment

**Fig. 3:**
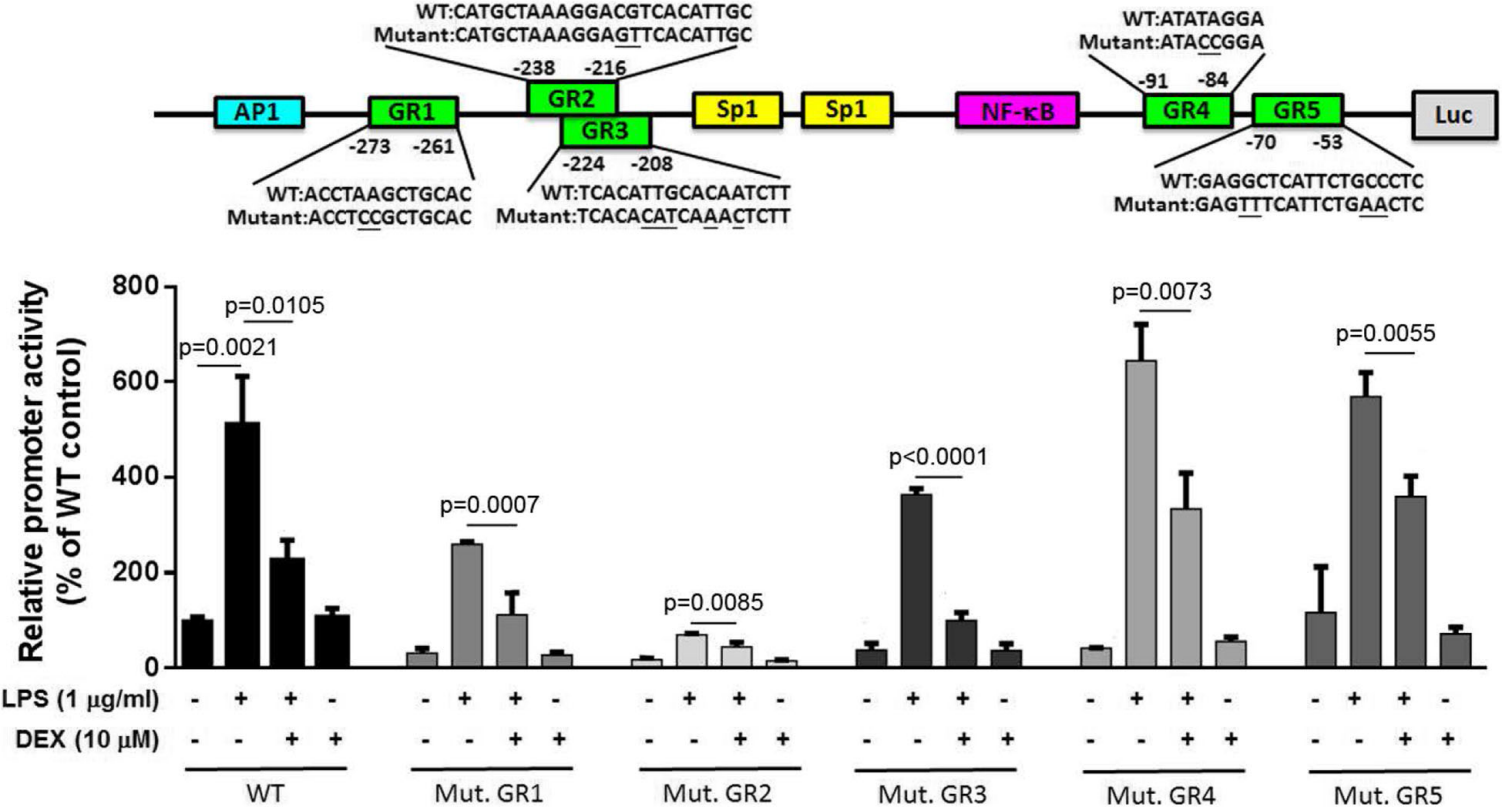
Mutation of putative GR2 binding site reduces the basal promoter activity of IL-6 gene. Upper reveals the mutant sequences of the five GR binding sites. Lower reveals the promoter activities of mutant GR sites. Among these, the mutant GR2 binding site revealed a significant decrease than the other mutant binding sites. All values are expressed as the mean ± SD and each experiment was conducted with triplicate cultures. The intergroup comparisons were performed using Student’s two-tailed unpaired *t*-test

Five putative GR binding sites were determined on the IL-6 promoter. We transfected the mutant promoter constructs of GR sites to detect the sites that are crucial for IL-6 promoter activities, and the results revealed that the mutation of GR2 site reduced the basal and LPS-induced promoter activities. Furthermore, the DEX-reversed effects were observed to have been altered. However, the mutations of GR1, GR3, GR4, and GR5 sites did not alter the effects of LPS and DEX treatments (Fig. 3). These results suggest that GR2 is crucial for IL-6 promoter activities.

### GRs bind on IL-6 promoter region

**Fig. 4:**
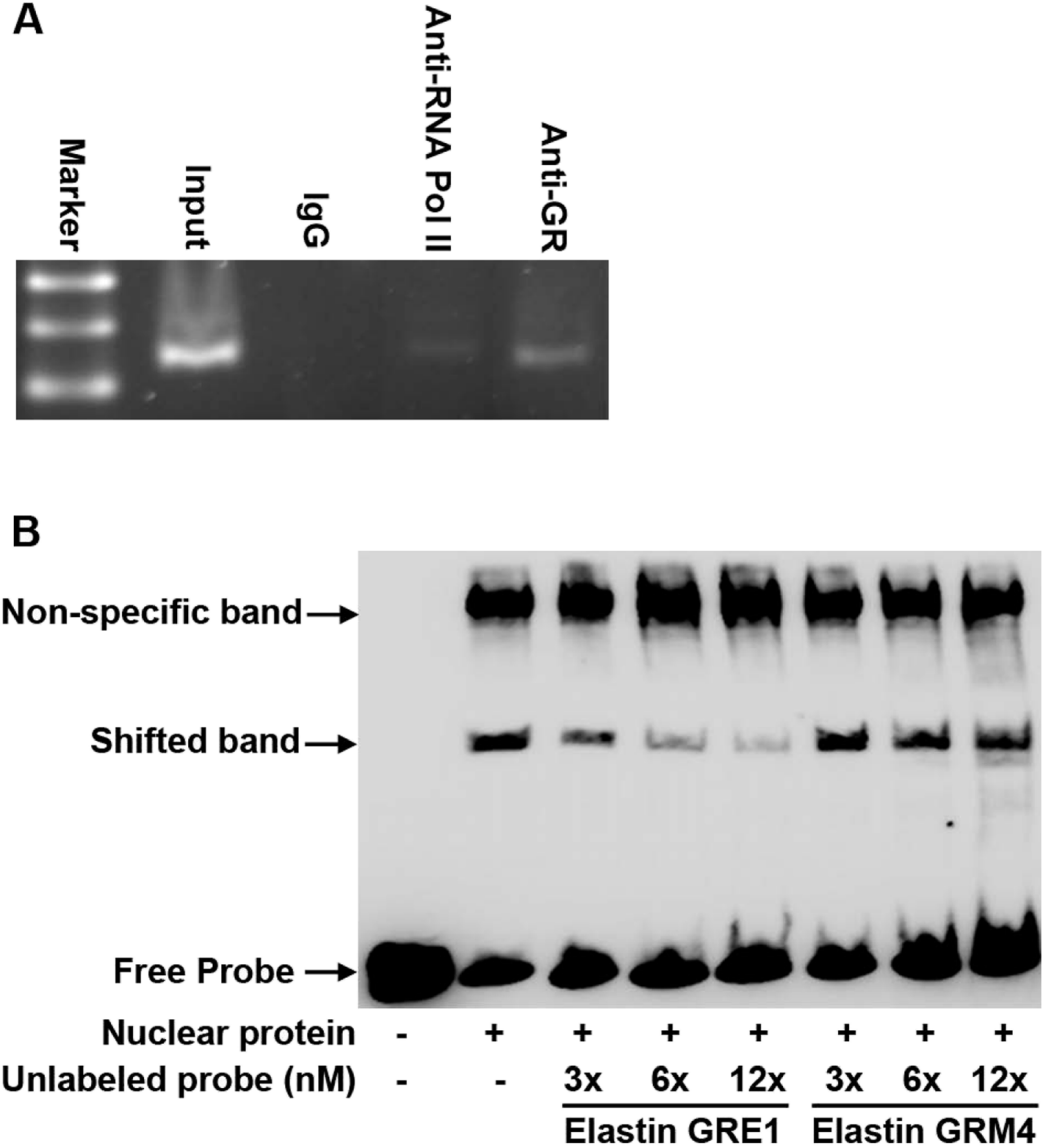
Glucocorticoid receptor (GR) directly binds to the IL-6 promoter and contributes to its activity. **a** GR binds to the IL-6 promoter region in vivo. Anti-mouse IgG and anti-RNA polymerase II antibodies were used as negative and positive controls in the ChIP assay, respectively. **b** Competition assay presents the GR binding on GR2 and GR3 sites of the IL-6 promoter. The fragments containing wild type and mutant GR binding motifs were used as competitors

To determine if GRs bind to the IL-6 promoter, we used ChIP and EMSA to investigate protein–DNA binding in vitro and in vivo, respectively. The findings of the ChIP assay revealed that GRs could bind to the IL-6 promoter (Fig. 4a). During the EMSA assay, the sequence analysis revealed that GR2 and GR3 overlapped partially; hence, we used one probe containing these two sites for EMSA. The EMSA results revealed that the probe of GR2 and GR3 sites exhibited shifted bands but not the other sites. Furthermore, we used the GR binding motif of the elastin promoter as cold probes for the competition assay and noted that the shifted bands were eliminated (Fig. 4b), suggesting that GR binds to GR2/3 sites. However, no shifted bands were noted in EMSA when using the probes containing GR1, 4, and 5 sites. Based on these results, we confirmed that GR binds to GR2/3 sites and not the others on the IL-6 promoter region.

### The TLR4 receptor downstream pathways were involved in LPS-induced and DEX-reversed IL-6 expression

**Fig. 5:**
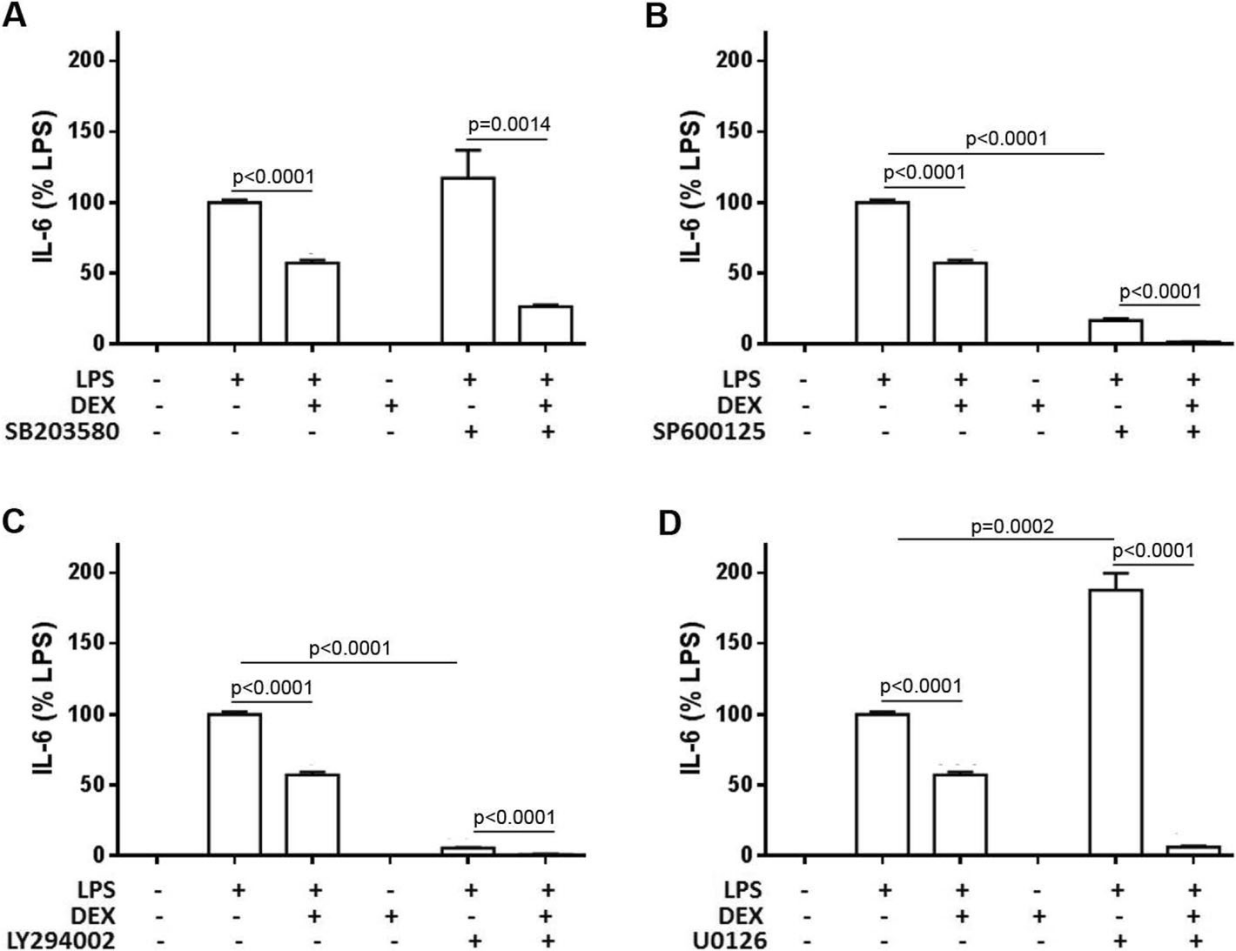
TLR4 downstream kinases are involved in lipopolysaccharide (LPS)-induced and dexamethasone (DEX)-reversed IL-6 secretion. The cells were treated with LPS, DEX and kinase inhibitors for 24 h, and then, the culture supernatants were harvested for measuring the concentrations of IL-6 using ELISA. **a** P38 MAPK inhibitor SB203580 (20 μM), **b** JNK inhibitor SP600125 (10 μM), **c** PI3K inhibitor LY294002 (10 μM) and **d** ERK inhibitor U0126 (10 μM). All values are expressed as the mean ± SD and each experiment was conducted with triplicate cultures. The intergroup comparisons were performed using Student’s two-tailed unpaired *t*-test

To further investigate the TLR4 receptor downstream pathways involved in IL-6 expression under LPS and DEX treatment, we treated cells with several kinase inhibitors (Fig. 5). We found that LPS induced IL-6 secretion and the inhibitors of JNK and PI3K but not P38 inhibited this effect, whereas, ERK promoted the IL-6 secretion. Treatment of DEX reduced the LPS-induced IL-6 secretion and these four inhibitors potentiated the DEX effects. These results indicated that TLR4 downstream kinases played differential roles in LPS- and DEX-mediated IL-6 production.

### The proposed schematic model for DEX regulates LPS-stimulated IL-6 expression through GRs

**Fig. 6:**
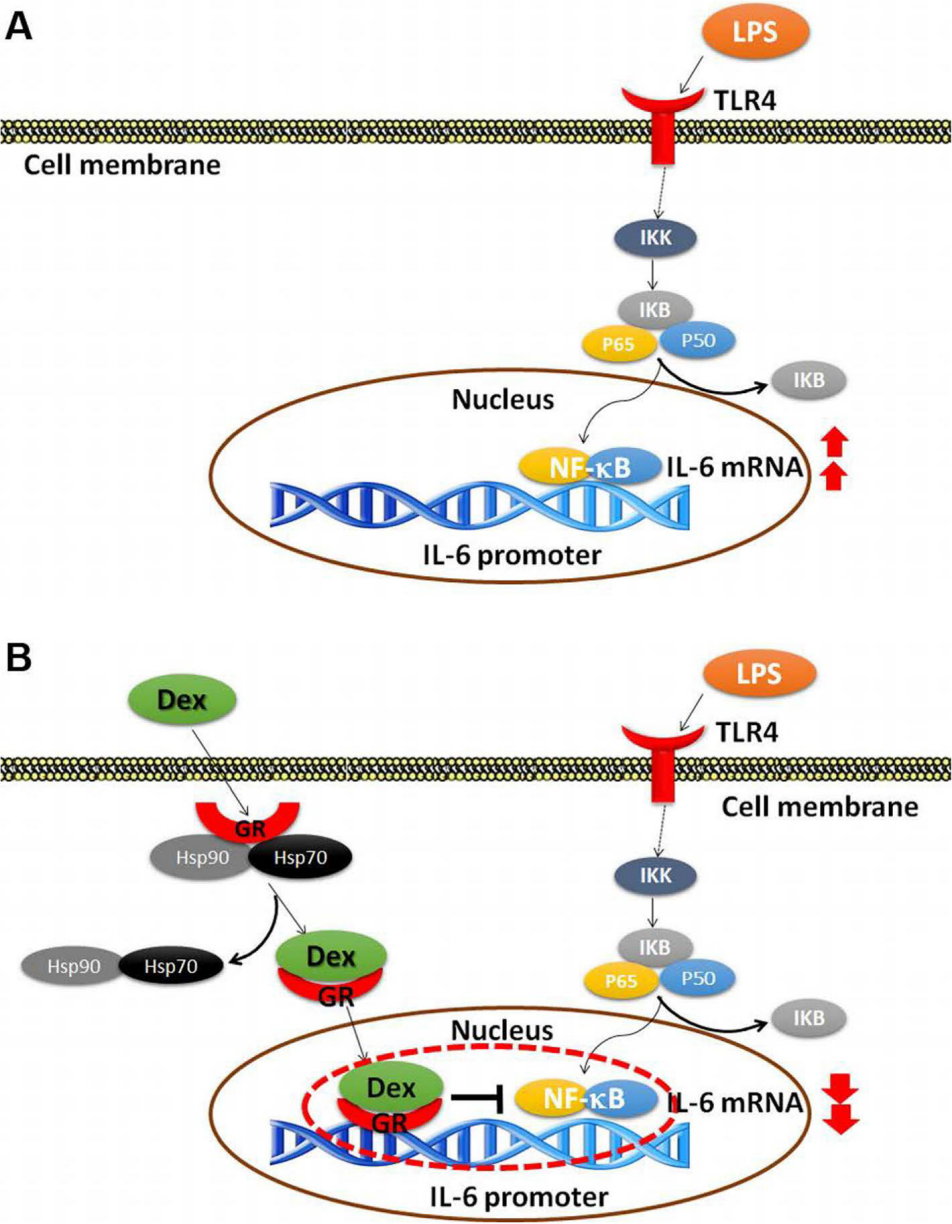
The schematic model for regulation of dexamethasone (DEX) in lipopolysaccharide (LPS)-stimulated IL-6 expression. **a** LPS stimulates IL-6 promoter activity through the NF-κB pathway in RAW 264.7 cells. **b** The proposed mechanism for counter-regulation of NF-κB by DEX and GR on the IL-6 promoter

We proposed a schematic model for DEX negatively regulating LPS-stimulated IL-6 expression in RAW 264.7 cells (Fig. 6). LPS stimulates nuclear IL-6 promoter activity through the NF-κB pathway, and a counter-regulation of NF-κB by the GR was proposed on the IL-6 promoter.

## Discussion

Our study results revealed that the pretreatment of RAW 264.7 cells with various concentrations of DEX could significantly reduce the LPS-induced mRNA expression of IL-6 in a dose-dependent manner. A protective role of synthetic DEX was indicated that affects LPS-induced inflammation. Our results are consistent with the findings of Yamazaki et al. who reported that GCs inhibit the synthesis of IL-1, TNF-α, IL-1β, IL-6, MMP-1, and COX-2 mRNA expression in SW 982 cells [22].

The molecular mechanism of GCs, wherein they interact with their GRs to regulate inflammatory responses, has been well-studied. GCs bind to GRs and are transported to the nucleus subsequently to form a dimer and combinations of transcriptional complexes, such as AP-1 and NF-κB, either in transrepression or transactivation, which are responsible for the anti-inflammatory action of GCs [23, 24]. The results of previous studies have suggested that the treatment of naïve monocytes with fluticasone or DEX did not cause global suppression of activated monocytic functions instead induced a cellular differentiation with an anti-inflammatory phenotype [25, 26]. The recent evidence revealed that GCs could efficiently inhibit these processes by downregulating pro-inflammatory mediators from macrophages and monocytes and their migration toward inflammatory stimuli. In addition, GCs could remove endo- and exogenous danger signals by increasing phagocytic capacity and limiting T-cell activation [27].

It has been known that LPS induces cytokine expression, such as IL-6, through TLR4 downstream pathways (Fig. 5&6). Several kinases are involved in these pathways, however, the mechanism are controversial [28–32]. We blocked the JNK activities decreased the LPS-induced IL-6 secretion and these results are consistent with a recent report [29]. In our study, we treated the Raw246.7 cells with P38 inhibitor**,** SB203580 was found that it did not change the secretion of IL-6 and these results are consistent with the previous report [31]. However, several lines of evidence showed that SB203580 reduced the LPS-induced IL-6 expression [29, 30]. When co-treating SB203580 with LPS and DEX, the levels of LPS-induced IL-6 secretion was further decreased than treatment of DEX. We also used LY294002 to inhibit PI3K and found that the LPS-induced IL-6 production was blocked, which is consistent with the previous report [32]. However, another report showed that LY294002 did not reduce IL-6 production [31]. We further investigated the role of ERK in IL-6 production and found that U0126 increased LPS-induced IL-6 production in the Raw246.7 cells. However, these results are different from other reports in which the authors found that U0126 inhibited LPS-induced effects [28, 31]. U0126 also enhanced the DEX effects to reduce the LPS-induced IL-6 production to very low level. According to these results, the roles of TLR4 downstream kinases under LPS and DEX treatment are controversial and remain to be elucidated. TLR4 signaling pathways involved in LPS- and DEX-mediated IL-6 expression remain further studied.

Our results revealed that LPS stimulation resulted in the upregulated expressions of inflammatory transcription factors, such as NF-κB [33], AP-1 [34], and Sp1–2 [35]. In addition, LPS increased IL-6 and its promoter activity. The increase of LPS-stimulated IL-6 promoter activities could be suppressed using DEX, especially in NF-κB, AP-1, and Sp1–2, but not in Sp1–1. Regarding to the inhibition efficiency, transcription factors plays differential roles in regulating IL-6 expression. NF-κB is the most important one, because of participating in basal promoter activity of IL-6 gene expression. The AP-1 and the second Sp1 sites are also involved in IL-6 expression, whereas, its importance is less than NF-κB. These results are consistent with those of previous studies [36, 37]. A previous study revealed two Sp1 sites on the IL-6 promoter and Sp1 bind to this region in human monocytes [38]. However, no reports were available to determine which Sp1 site was crucial for IL-6 expression. Our study is the first to report that only the Sp1–2 site is involved in IL-6 expression. Conclusively, the transcription factors NF-κB, AP-1, and Sp1–2 bind to the IL-6 promoter and are playing crucial roles in the basal and inducible expression of murine IL-6 gene.

Our study determined five putative GR binding sites on the IL-6 promoter, and DEX could reduce the promoter activities of IL-6 GR binding sites. After LPS stimulation and sequence construct, the promoter activity of IL-6 exhibited a significant increase in LPS-induced promoter activity. Notably, GR2 site appeared to play a crucial role in both basal and inducible promoter activities in LPS-induced inflammation. By contrast, the GR1, GR3, GR4, and GR5 sites did not exhibit changes in LPS-induced or basal promoter activities. Although a previous study revealed that the recombinant GR binds to IL-6 promoter regions, namely the GR1–5 elements, in vitro [12], no significant shifted bands were noted using the probes of GR1, GR4, and GR5 during EMSA, and no changes of promoter activities of LPS- and DEX-involved effects were observed after the mutation of these three sites. Furthermore, GRs may interact with transcription factors, such as AP-1 and NF-κB, to mediate downstream proinflammatory genes, including IL-6 [39]. Nevertheless, the precise targeted regulation of GR2 on IL-6 and the possible mechanism related to transcriptional synergism with the GC response element (GRE) remain unknown. Nonetheless, additional studies are warranted to determine whether GR2 represents a novel negative GRE [40], directly binding through GRα—the pivotal subunit of GR—that could facilitate interactions between GRα and AP-1 and NF-κB.

Recently, the RECOVERY (Randomized Evaluation of COVID-19 ThERapy) trial showed a striking result that DEX could reduce nearly one-third deaths of critically ill patients receiving respiratory support [41, 42]. The coronavirus (SARS-CoV2) could not only damage lung alveolar cells but also induce high levels of macrophage-related cytokines, IL-6, IL-10, and TNF-α in severe cases, demonstrated that the overactive immune response could be effectively or partially suppressed by DEX in this coronavirus pandemic [43].

## Conclusions

In summary, our results provide possible insights into the mechanism of controversial steroids that would help with the decision-making of their dosages—low or high—in hemodynamically unstable patients with sepsis. Moreover, in the future, knowledge regarding GR2 binding site could prove crucial in facilitating the selection of individual-specific therapeutic agents in vulnerable patients with sepsis or SARS-CoV2 infection.

## Supplementary Information

**Additional file 1.**

**Additional file 2.**

## Data Availability

All data generated or analyzed during this study are included in this published article [and its supplementary information files].

## Abbreviations

GCs: Glucocorticoids
GR: GC receptor
GRE: GC response element
GILZ: Glucocorticoid-induced leucine zipper
IL-6, 8, 12: Interleukin-6, 8, 12
DEX: Dexamethasone
LPS: Lipopolysaccharide
NF-κB: Nuclear factor-kappa В
AP-1: Activator protein-1
Sp1–2: Specificity protein 1–2
TLRs: Toll-like receptors
MIF: Migration inhibitory factor
EMSA: Electrophoretic mobility shift assay
RT-PCR: Real-time-polymerase chain reaction
ChIP: Chromatin immunoprecipitation
