# Correction to: Mutant glucocorticoid receptor binding elements on the interleukin-6 promoter regulate dexamethasone effects

**DOI:** 10.1186/s12865-021-00437-5

**Published:** 2021-07-12

**Authors:** Wen-Teng Chang, Ming-Yuan Hong, Chien-Liang Chen, Chi-Yuan Hwang, Cheng-Chieh Tsai, Chia-Chang Chuang

**Affiliations:** 1 Department of Biological Science and Technology, Chung Hwa University of Medical Technology, Tainan, 701 Taiwan; 2 Department of Emergency Medicine, National Cheng Kung University Hospital, College of Medicine, National Cheng Kung University, Tainan, Taiwan; 3 Department of Physical Therapy, I-Shou University, Kaohsiung, Taiwan; 4 Department of Nursing, Chung Hwa University of Medical Technology, Tainan, 701 Taiwan

**Correction to: BMC Immunol 22, 24 (2021)**

**DOI: 10.1186/s12865-021-00413-z**

In this article [1] in the Ethics approval and consent to participate section the IRB was incorrectly given as (IRB No: B-ER-102-121 to CCC). but should have been (IRB No: A-ER-102-200 to CCC). The original article has been updated.
