# Supplementary material for: Mutant glucocorticoid receptor binding elements on the interleukin-6 promoter regulate dexamethasone effects
Source: BMC Immunol. 2021 Mar 26;22:24. doi: 10.1186/s12865-021-00413-z (PMC7995394; doi:10.1186/s12865-021-00413-z)
Supplement: Supplementary file 1 — Additional file 1. [file 12865_2021_413_MOESM1_ESM.pdf]

```
Human   CAAGACATGCCAAAGTGCTGAGTCACTAATAAAGAAAAAAAGAAAGTAAAGGAAGAGTGGTTCTGCTTCTTAGCGCTAGCCTCAATGACGACCTAAGCT -265
Monkey  CAAGACATGCCAAAGTGCTGAGTCACTAATAAAGAAAAAAAGAAAGTAAAGGAAGAGTGATTCTGCTTCTTAGCGCTAGCCTCAGTGACGACCTAAGCT -262
Horse   CAAGACGTGCCAAAATGCTGAGTCACAA--AAAAAAGGAAAAGAAAGTAA-------GTGA----GCTTCTTAGGGCTACGCTT-GTAATGACCTAAGCG -265
Mouse   CAAGACATGCTCAAGTGCTGAGTCACTTTTAAAGAAAAAAAAGAAGA----------GTGCTCATGCTTCTTAGGGCTAGCCTCAAGGATGACTTAAGC- -257
Rat     CAAG---TGCTCAAGTGCTGAGTCACTTTTAAAGAAAGAAA--AAGA----------GTGATCAGGCTTCTTAAGGATAGCCTCAAGGATGACTTAAAC- -267
        ****   ***  ** ***********   ****   ***  ** ***********    *** *** *  ******  **   **   * ** *  ****

AP-1   GR1

Human   GCACTTTTCCC--CCTAGTTGTG--TCTTGCCATGCTAAAGGACGTCACATTGCACAATC-TTAATAAGGTTTCCAATCAGCCCCACCCGCTCTGGCCCC -170
Monkey  GCACTTTTCCC--CCTAGTTGAG--TCTTGCGATGCTAAAGGACGTCACATTGCACAATC-TTAAT---GTTTCCAATCAGCCCCACCCGCTCTGGCCCC -170
Horse   GCACTTTTTCC--CC-GGTTGTGATTCTTGTGATGCTAAAGGACGTCACATTGCACAATCCTTAATAAGGTTTCCAATCAGCCCCACCCGCTCTGGCCCC -168
Mouse   ACACTTTCCCCTTCCTAGTTGTGATTCTTTCGATGCTAAACGACGTCACATTGTGCAATC-TTAATAAGGTTTCCAATCAGCCCCACCCACTCTGGCCCC -158
Rat     ACACTTTCCCCCTCCTAGCTGTGATTCTTTGGATGCTAAATGACGTCACATTGTGCAATC-TTAATAAGGTTTCCAATCAGCCCCACCCACTCTGGCCCC -168
         ******   **   **   * ** *   ****    ******** ***********  ***** *****    ******************* **********

GR2/CREB   GR3/NF-1L6   Sp1   Sp1

Human   ACCCTCACCCTCCAACAAAGATTT--ATCAAATGTGGGATTTTCCCATGAGTCTCAATATTAGAGTCTCAACCCCCAATAAATATAGGACTGGAGATGTC -72
Monkey  ACCCTAACCCTCCAACAAAGATTT--ATCAAATGTGGGATTTTCCCATGAGTCTCAATATTAGAGTCTCAACCCCCAATAAATATGAGACTGGAGATGTC -72
Horse   ACCCCCACCCTCCAACAAAGATTT--ATCAAATGTGGGATTTTCCCATGAGTCTCAATATTAGAGTCTCAACCCCCAATAAATATGAGACTGGGATGTC -70
Mouse   ACCCCCACCCTCCAACAAAGATTTTTATCAAATGTGGGATTTTCCCATGAGTCTCAAAATTAGAGAGTTGACTCCTAATAAATATGAGACTGGGATGTC -58
Rat     ACCCCCACCCTCCAACAAAGATTTTTATCAAATGTGGGATTTTCCCATGAGTCTCAAAAGTAGAGAGTCGACTCCCAATAAATATGAGACTGGGATGTC -68
        ****  ******************   ******************************* * *****   *   ** ** *********  ****** ******

NF-κB   GR4

Human   TGAGGCTCATTCTGCCCTCGAGCCCACCGGGAACGAAAGAGAAGCTCTATCTCCCCTCCAGGAGCCCAGCTATGAACTCCTTCTCCACAA +19
Monkey  TGAGGCTCATTCTGCCCTCGAGCCCACCAGGAACGAAAGAGAAGCTCTATCTGCCCTCCAGGAACCCAGATATGAACTCCGTCTCCACAA +19
Horse   TGAGGCTCATTCTGCCCTCGAGCCCACCAGGAACGAAAGAGA-GCTTCATCTGCCCTCCAG-AACCCAGCTATGAACTCCCTCTTCACAA +19
Mouse   TGTAGCTCATTCTGCTCTGGAGCCCACCAAGAACGATAGTCA-ATTCCA-----------GAAACC--GCTATGAAGTTCCTCTCTGCAA +19
Rat     TGTAGCTCATTCTG-TCTCGAGCCCACCAGGAACGAAAGTCA-ACTCCATCTGCCCTTCAGGAACA--GCTATGAAGTTTCTCTCCGCAA +19
        **  **********  ** *********   ****** **  *   *  *          * * *   * ****** *   ***   ***

GR5
```
