# Supplementary material for: Mutant glucocorticoid receptor binding elements on the interleukin-6 promoter regulate dexamethasone effects
Source: BMC Immunol. 2021 Mar 26;22:24. doi: 10.1186/s12865-021-00413-z (PMC7995394; doi:10.1186/s12865-021-00413-z)
Supplement: Supplementary file 2 — Additional file 2. [file 12865_2021_413_MOESM2_ESM.docx]

*** Supplemental information**

Table 1. Primers used in this study.

| Primers for RT-PCR | |
| --- | --- |
| mIL6-mF1 | CAAAGAAATGATGGATGCTACC |
| mIL6-mR1 | ATTGGAAATTGGGGTAGGAAGG |
| Primers for cloning wild type IL-6 promoter fragment | |
| IL6-F | GGGGTACCCAAGACATGCCAAAGTGCTGAGT |
| IL6-R | CCCAAGCTTGGGGAGATAGAGCTTCTCTTTCG |
| Primers for cloning mutant GR binding sites on IL-6 promoter | |
| IL6-GR1-F | CTAGCCTCAATGACGACAGAATCTGCACTTTTCCC |
| IL6-GR1-R | GGGAAAAGTGCAGATTCTGTCGTCATTGAGGCTAG |
| IL6-GR2-F | CTTGCCATGCTAAAGGAGTTCACATTGCACAATC |
| IL6-GR2-R | GATTGTGCAATGTGAACTCCTTTAGCATGGCAAG |
| IL6-GR3-F | CTAAAGGACGTCACACATCAAACTCTTAATAAGGTTTCC |
| IL6-GR3-R | GGAAACCTTATTAAGAGTTTGATGTGTGACGACCTTTAG |
| IL6-GR4-F | CCCCCAATAAATACCGGACTGGAGATGTCTGAGGC |
| IL6-GR4-R | GCCTCAGACATCTCCAGTCCGGTATTTATTGGGGG |
| IL6-GR5-F | GAGATGTCTGAGTTTCATTCTGAACTCGAGCCCACC |
| IL6-GR5-R | GGTGGGCTCGAGTTCAGAATGAAACTCAGACATCTC |
| IL-6-AP1-F | GACATGCCAAAGTGCTGCAGCACTAATAAAAGAAA |
| IL-6-AP1-R | TTTCTTTTATTAGTGCTGCAGCACTTTGGCATGTC |
| IL-6-NF-κB -F | ATCAAATGTGGGATTTTAGAATGAGTCTAATATTAGAG |
| IL-6-NF-κB -R | CTCTAATATTAGACTCATTCTAAAATCCCACATTTGAT |
| IL-6-Sp1-1-F | GGTTTCCAATCAGCCCCAAACGCTCTGGCCCCACCC |
| IL-6-Sp1-1-R | GGGTGGGGCCAGAGCGTTTGGGGCTGATTGGAAACC |
| IL-6-Sp1-2-F | CACCCGCTCTGGCCCCAAACTCACCCTCCAACAAAG |
| IL-6-Sp1-2-R | CTTTGTTGGAGGGTGAGTTTGGGGCCAGAGCGGGTG |
| Primers for ChIP assay | |
| mIL6-cpF1 | TTCCCATCAAGACATGCTCAAG |
| mIL6-cpR3 | TTGATAAAAATCTTTGTTGGAGG |
| Primers for cloning GR cDNA fragment | |
| GR-1F | CTAGCTAGCATATTCACTGATGGACTCCAAAG |
| GR-2F | AATACAGCATCCCTTTCTCAACA |
| GR-3R | CTGTCCTTCCACTGCTCTTTTGA |
| GR-4R | GCTCTAGAAACCATTCTTATTAAGGCAGTCA |
| GR-5F | AAGTTGTTTATCTCGGCTG CGGC |
| GR-6R | CAAAACCTCTACAGGACAAACTG |
